# N-terminal Slit2 inhibits HIV-1 replication by regulating the actin cytoskeleton

**DOI:** 10.1186/1742-4690-10-2

**Published:** 2013-01-07

**Authors:** Appakkudal R Anand, Helong Zhao, Tirumuru Nagaraja, Lisa A Robinson, Ramesh K Ganju

**Affiliations:** 1 Department of Pathology, Ohio State University Wexner Medical Center, 460 W 12th Avenue, 810 Biological Research Tower, Columbus, OH, 43210, USA; 2 The Hospital for Sick Children, 555 University Ave, Room 5265, Toronto, ON, M5G 1X8, Canada

## Abstract

**Background:**

Slit2 is a ~ 200 kDa secreted glycoprotein that has been recently shown to regulate immune functions. However, not much is known about its role in HIV (human immunodeficiency virus)-1 pathogenesis.

**Results:**

In the present study, we have shown that the N-terminal fragment of Slit2 (Slit2N) (~120 kDa) inhibits replication of both CXCR4 and CCR5-tropic HIV-1 viruses in T-cell lines and peripheral blood T-cells. Furthermore, we demonstrated inhibition of HIV-1 infection in resting CD4+ T-cells. In addition, we showed that Slit2N blocks cell-to-cell transmission of HIV-1. We have shown that Slit2N inhibits HIV-1 infection by blocking viral entry into T-cells. We also ruled out Slit2N-mediated inhibition of various other steps in the life cycle including binding, integration and viral transcription. Elucidation of the molecular mechanism revealed that Slit2N mediates its functional effects by binding to Robo1 receptor. Furthermore, we found that Slit2N inhibited Gp120-induced Robo1-actin association suggesting that Slit2N may inhibit cytoskeletal rearrangements facilitating HIV-1 entry. Studies into the mechanism of inhibition of HIV-1 revealed that Slit2N abrogated HIV-1 envelope-induced actin cytoskeletal dynamics in both T-cell lines and primary T-cells. We further showed that Slit2N specifically attenuated the HIV-1 envelope-induced signaling pathway consisting of Rac1, LIMK and cofilin that regulates actin polymerization.

**Conclusions:**

Taken together, our results show that Slit2N inhibits HIV-1 replication through novel mechanisms involving modulation of cytoskeletal dynamics. Our study, thus, provides insights into the role of Slit2N in HIV-1 infection and underscores its potential in limiting viral replication in T-cells.

## Background

Slits belong to a group of large secretory glycoproteins that were initially described as regulating axonal guidance during the development of the central nervous system [1,2]. Slit consists of a family of three genes: Slit1, Slit2 and Slit3 that are highly homologous to each other and encode ligands for the Roundabout (Robo) receptors [3,4]. It is now clear that Slit and Robo genes are expressed in a range of tissues in addition to the brain, where Slit-Robo signaling has critical functions [5]. However, information on the effects of Slit2 in non-neuronal systems is not well-studied, with recent studies indicating that Slit2/Robo1 complex regulates lung and kidney development, tumor angiogenesis and metastasis [6-11]. With regard to the immune system, Slit2 has been shown to inhibit migration of hematopoietic cells, monocytes, neutrophils, dendritic cells, and T lymphocytes, toward chemoattractant signals [12-15]. Specifically, we and others have shown Slit2 blocks CXCL12/CXCR4-mediated chemotaxis in T-cells [15,16]. In addition, we recently showed that full-length Slit2 inhibited both X4 and R5-tropic HIV-1 replication in T-cells [17]. Recently, Slit2N also was shown to regulate HIV-1-gp120-induced endothelial permeability [18].

A prototypical Slit2 protein contains an N-terminal signal peptide, four leucine-rich repeats (LRRs), seven (in *Drosophila* Slit) or nine (in vertebrate Slits) EGF repeats, and a C-terminal cysteine knot [19]. Studies have shown that full-length Slit2 is cleaved between the fifth and sixth EGF-like repeat into a 120–140 kDa N-terminal and a 50–60 kDa C-terminal fragment [20]. Recent evidence suggests that the N-terminal region of Slit2 (Slit2N) is responsible for the biological functions of Slit2 [5,21].

The intracellular signal transduction mechanism for Slit2/Robo1 signaling is not well-studied. However, several lines of recent evidence have demonstrated that Slit2 regulates actin polymerization after binding to Robo receptor [16,22-24]. Robo is a transmembrane receptor that consists of a fibronectin type III and immunoglobulin (Ig)-like domains and an intracellular cytoplasmatic domain. The intracellular domain of Robo has been shown to interact with proteins that regulate the Rho family of small guanosine triphosphates (GTPases), which play well-defined roles in cell migration, cytoskeletal organization and remodeling by eliciting changes in actin cytoskeleton [25]. Furthermore, HIV has the capacity to bind to its receptors, CD4 and/or co-receptors (CXCR4 or CCR5) and induce signal transduction pathways that trigger actin cytoskeletal rearrangements facilitating viral entry [26-30].

In the present study, we have analyzed the effect of N-terminal domain of Slit2 (Slit2N) in HIV-1 infection and shown that it inhibits replication of both X4 and R5-tropic HIV-1. Furthermore, our mechanistic studies in T-cell lines and primary T-cells have revealed that Slit2 inhibits the HIV-1 viral entry through a novel mechanism involving modulation of actin cytoskeletal dynamics.

## Results

### N-terminal fragment of Slit2 mediates anti-HIV-1 activity

**Figure 1:**
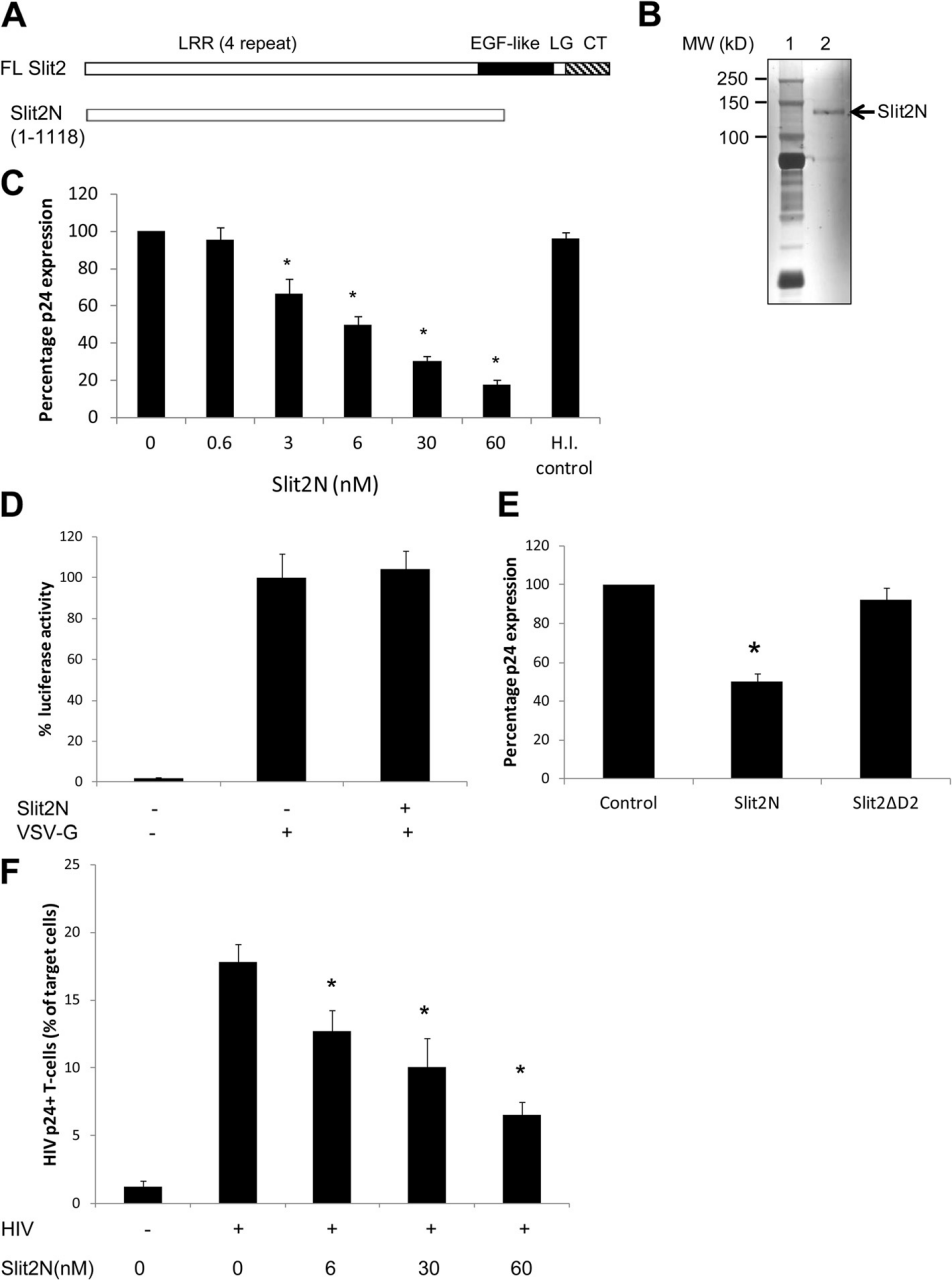
**The N-terminal Slit2 fragment possesses anti-HIV-1 activity.** (**A**) Domain organization of Slit2 showing LRR, leucine-rich repeat; EGF, epidermal growth factor-like; LG, laminin G-like; CT, C-terminal cystine regions. (**B**) Silver staining of the Slit2N protein. (**C**) MT4 cells pre-treated with various concentrations of Slit2N were infected with HIV-1 IIIB (10 ng p24). We used heat-inactivated (H.I) Slit2N as a control. After 48 hours, supernatants were collected for estimation of HIV-1 p24 antigen levels by ELISA. Virus production in the positive control (control HIV-1 infected MT4 cells): 4.8 ng/ml p24 Ag. Heat-inactivated Slit2N at a concentration of 60nM was used as a control. (**D**) MT4 cells were treated with Slit2-N (6 nM) for 30 minutes and then infected with HIV-Luc viruses pseudotyped with VSV-G envelope (VSV-G) for 2 hours. Forty-eight hours post infection, cells were harvested for the Luciferase assay. (**E**) MT4 cells were treated with equivalent concentrations of Slit2N and Slit2 ΔD2 (≈6nM) and infected with HIV-1 IIIB (10 ng p24) for 48 hours. Virus production in the supernatants was estimated by HIV-1 p24 ELISA. (**F**) MT4 cells infected with HIV-1 IIIB were co-cultured with dye-labeled target Jurkat T-cells in the presence of various concentrations of Slit2N for 4 hours, and the percentage of Gag^+^ target cells was measured by flow cytometry. The results are represented in a bar graph. Values are the mean percentages of Gag^+^ dye-labeled target cells with the SEMs. Infected cells were used when >70-80% of the donor cells were routinely Gag^+^ by flow cytometry. Data are representative of three independent experiments. *P ≤ 0.05 versus untreated cells.

Slit2 contains the N-terminal region consisting of four leucine-rich repeats (LRRs), nine epidermal growth factor (EGF) repeats, a laminin G domain, and a C-terminal cysteine-rich region (Figure 1A) [19]. Studies have shown that full-length Slit2 is cleaved between the fifth and sixth EGF-like repeat into a 120–140 kDa N-terminal and a 50–60 kDa C-terminal fragment, and the biological effects of Slit2 are mediated by the N-terminal fragment [20]. In the present study, we first analyzed the anti-HIV-1 activity of a purified Slit2N fragment.

Firstly, the purity of Slit2N was determined by silver staining (Figure 1B). To confirm that Slit2N does not affect the viability and proliferation of T-cells, we performed the MTT assay (Roche, Indianapolis, IN) as per the manufacturer’s recommendations, using various concentrations of Slit2N. We found that Slit2 did not affect cell viability or cell proliferation (data not shown). We also demonstrated that Slit2 did not affect T-cell activation (data not shown). Next, we evaluated the anti-HIV-1 activity of Slit2N against cell-free virus. We used a range of concentrations of Slit2N (0.6-60 nM), and Slit2N clearly showed a dose-dependent inhibition of HIV-1 replication in MT4 cells (Figure 1C). We obtained a maximum inhibition of 82.6% when Slit2N was used at a concentration of 60 nM.

To check the specificity of activity of Slit2N against HIV-1, we pre-incubated MT4 cells with or without Slit2N and then infected these cells with HIV-luc pseudotyped with the VSV-G envelope protein. This single-life cycle virus does not require HIV receptors to enter the target cells [31]. Incubation of MT4 cells with Slit2N had no effect on infection with HIV-Luc pseudotyped with VSV-G (Figure 1D).

Several recent studies have indicated that the functional effects of Slit2 are concentrated in the D2 domain of Slit2 [4,32]. To verify whether the D2 region is responsible for the anti-HIV activity of Slit2, we obtained a mutant Slit2 protein that lacks the second leucine-rich region that is necessary for binding to Robo1 (Slit2ΔD2) [33]. MT4 cells were infected with HIV-1 IIIB in the presence of equivalent concentrations of Slit2N and Slit2 ΔD2 (≈6nM). We found that Slit2 ΔD2 showed no inhibition of HIV replication in comparison to Slit2N (Figure 1E), indicating that the anti-HIV activity of Slit2 is restricted to the D2 domain of Slit2.

In addition to infection with cell-free virions, the importance of cell-associated spread across connecting membrane bridges and close cell–cell contacts is increasingly recognized and thought to constitute the predominant mechanism of HIV-1 propagation in vivo [34,35]. We, therefore, examined whether Slit2N has an effect on cell-cell transmission of HIV-1. Briefly, MT4 cells were first productively infected with HIV-1 IIIB. After 24 hours, ~70-80% of the cells were HIV-1 p24 positive by intracellular p24 staining and flow cytometry (data not shown). These cells were used as donors and co-cultivated with Far-red Cell trace dye-labeled Jurkat T-cells in the presence of various concentrations of Slit2N, as described in the Methods. As shown in Figure 1F, we clearly observed a dose-dependent inhibition of HIV infection in the target cells with maximum inhibition of 62.5% when Slit2N was used at 60nM. These studies indicate that Slit2N also inhibits cell to cell spread of HIV in T-cells.

**Figure 2:**
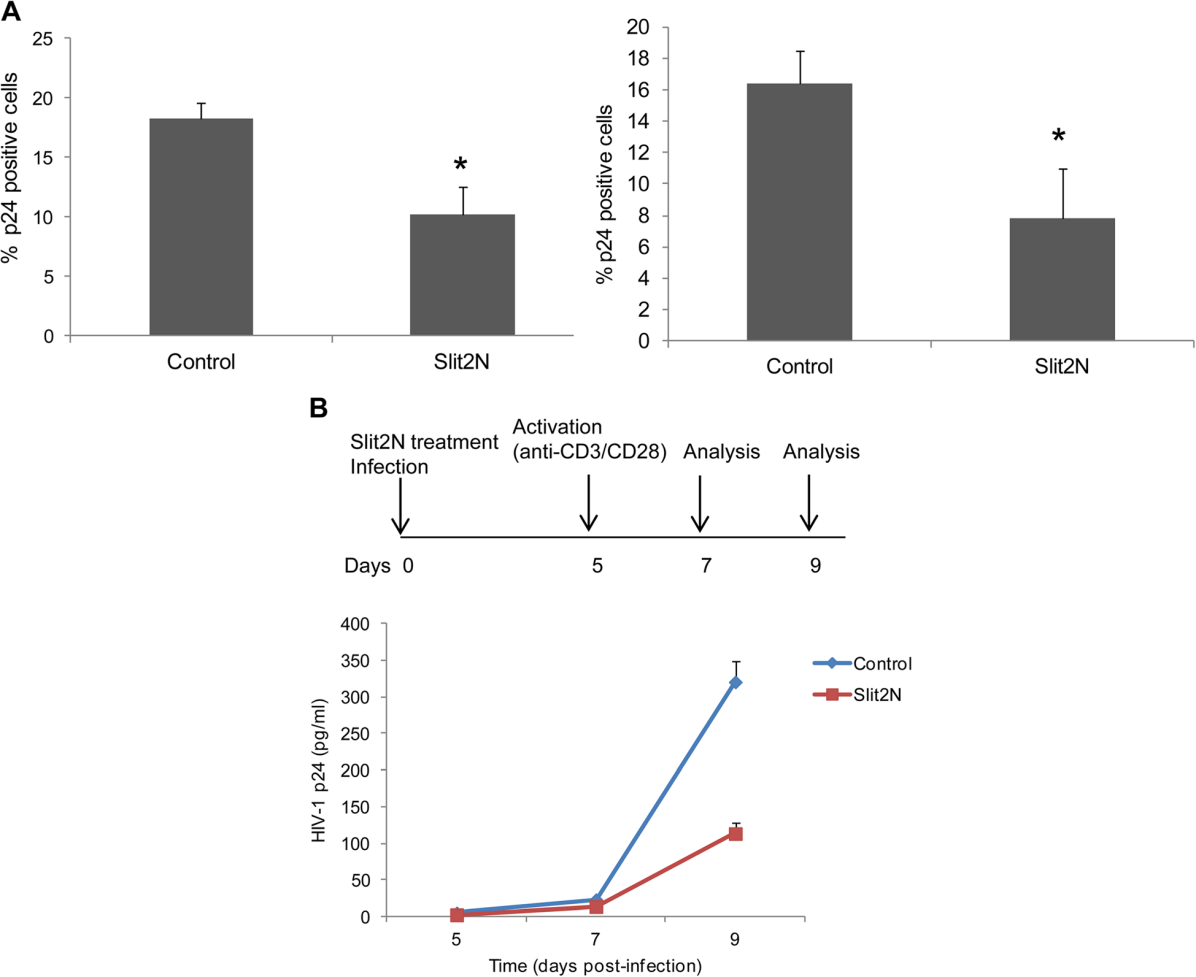
**Slit2N inhibits HIV infection of activated and resting CD4**^**+**^**T-cells.** (**A**) PHA-stimulated CD4^+^ T-cells were infected with HIV-1 IIIB (left panel) or HIV-1 BaL (right panel) in the presence of Slit2N (3nM). The cells were incubated in IL-2 containing medium, and the cells were collected after 48 hours and analyzed for HIV-1 p24 expression by flow cytometry. *P < 0.05 versus untreated cells infected with HIV-1. (**B**) Resting CD4^+^ T-cells were infected with HIV-1 IIIB in the presence or absence of Slit2N (3nM). Following infection, the cells were washed to remove unbound virus and cultured in the presence of Slit2N for 5 days. Cells were activated at day 5 with anti-CD3/CD28 magnetic beads, and viral replication was measured by p24 release. Results are representative of 3 separate experiments.

We further evaluated the effect of Slit2N on HIV-1 IIIB (X4) and BaL (R5) replication in PHA-stimulated CD4^+^ T-cells. We observed an approximately 40% reduction in HIV-1 replication with both X4-tropic virus and R5-tropic virus (Figure 2A, left and right panel), indicating that Slit2N inhibits HIV-1 replication irrespective of the co-receptor used by the virus. We also tested the effect of Slit2N on infection of resting CD4+ T-cells. Cells pre-treated with Slit2N and infected with HIV-1 IIIB were incubated for 5 days, after which they were activated with anti-CD3/CD28 beads to initiate viral replication (Figure 2B, upper panel). Supernatants collected at various time points after activation were examined for HIV-1 p24 levels by ELISA. We observed a significant reduction in HIV infection (approximately 65%) in resting CD4+ T-cells (Figure 2B, lower panel).

### Slit2 attenuates viral entry

**Figure 3:**
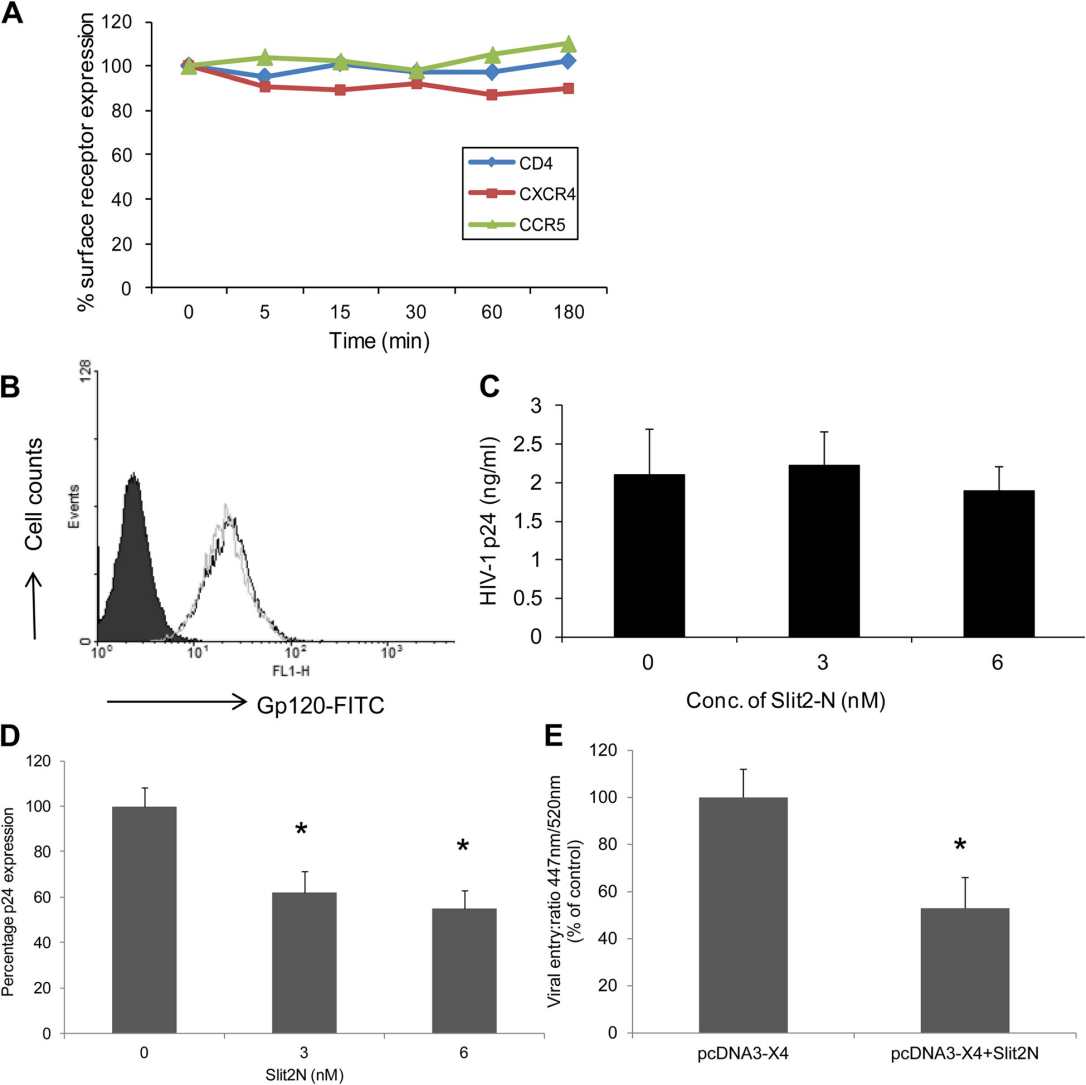
**Slit2N blocks HIV-1 entry, but does not affect HIV-1 receptor expression, envelope binding or HIV-1 virus binding to T-cells.** (**A**) To analyze the effect of Slit2N on HIV-1 receptor expression, MT4 cells or PBMCs were treated with Slit2N for various time periods. MT4 cells were analyzed for CD4 and CXCR4 expression while PBMCs were analyzed for CCR5 expression, respectively, by flow cytometry. (**B**) MT4-cells were treated with Slit2N and analyzed for Gp120 binding by flow cytometry using FITC-tagged gp120. (**C**) For virus binding, untreated and Slit2N (3 nM and 6 nM) -treated MT4 cells were infected with HIV-1 IIIB (40 ng/10^6^ cells) and incubated for one hour at 4°C, and the cell lysates were analyzed for HIV-1 p24 levels. (**D**) MT4 cells infected with HIV-1 IIIB were incubated for 3 hours at 37 ° C in the presence or absence of Slit2N (3nM and 6nM). The cells were treated with trypsin, washed, lysed and analyzed for intracellular p24 antigen levels by ELISA. *P < 0.05 versus the control preparation. (**E**) Untreated and Slit2N-treated MT4 cells were incubated with BlaM-Vpr-containing virions (500 ng of p24), after which the cells were washed and incubated with CCF2-AM loading mix (GeneBLAzer detection kit; Invitrogen). The excess dye was washed off and cells were incubated for 16 h at room temperature before fixation with 4% paraformaldehyde. The entry of Blam-Vpr containing virions was measured as the ratio of the maximum fluorescence intensity between cleaved and intact CCF2. *P < 0.05 versus the control infected cells. Results are representative of 3 separate experiments.

In order to determine how Slit2N-mediated inhibition works, we first determined the step in the HIV-1 life cycle that was inhibited by Slit2N. Time of addition assays pointed to the inhibition of early pre-integrative steps in the viral life cycle (data not shown). Our next objective was to further determine the exact pre-integrative step in the viral life cycle that is inhibited by Slit2N. The first step in the viral life cycle is the binding of the virus onto the receptors on the T-cells. Therefore, we first determined whether HIV-1 receptor expression was affected by Slit2N. Treatment of cells with Slit2N did not affect membrane expression of CD4, CXCR4 or CCR5, which are required for HIV-1 binding and entry into cells (Figure 3A). To investigate whether Slit2N is capable of inhibiting the binding of HIV-1 gp120 to its receptors on T-cells, we analyzed the binding ability of FITC-conjugated recombinant gp120 to the Slit2N-treated and untreated MT4 cells. As shown in Figure 3B, Slit2N did not affect the binding of gp120 to T-cells. We also determined the binding of HIV-1 virus to MT4 cells using the virus binding assay. As shown in Figure 3C, though there was no difference in the binding of HIV-1 onto the T-cells. Together, these results indicated that the binding of virus to the receptors on T-cells was not affected by Slit2N.

Next, we further specifically tested the ability of Slit2N to block cellular entry of HIV-1 in MT4 cells using a HIV-1 virus entry assay. We observed a significant reduction (~40%) in the amount of virus that enters the cell in Slit2N-treated cells as compared to the control (Figure 3D). To further analyze the role of Slit2N in inhibiting HIV-1 infection, we performed viral fusion and entry experiments by using X4-tropic HIV-1 viral particles containing the BlaM-Vpr chimera [36]. These chimera virions have been designed to specifically study the first steps of viral infection, since β-lactamase activity directly correlates with viral entry. Untreated and Slit2N-treated MT4 cells were incubated (3 h) with equivalent viral inputs of X4-tropic virions containing BlaM-Vpr fusion protein. Untreated cells were susceptible to viral entry, whereas in cells treated with Slit2N, viral entry was strongly reduced (~50%) (Figure 3E). Taken together, these data indicate that Slit2N inhibits HIV-1 viral fusion and entry.

**Figure 4:**
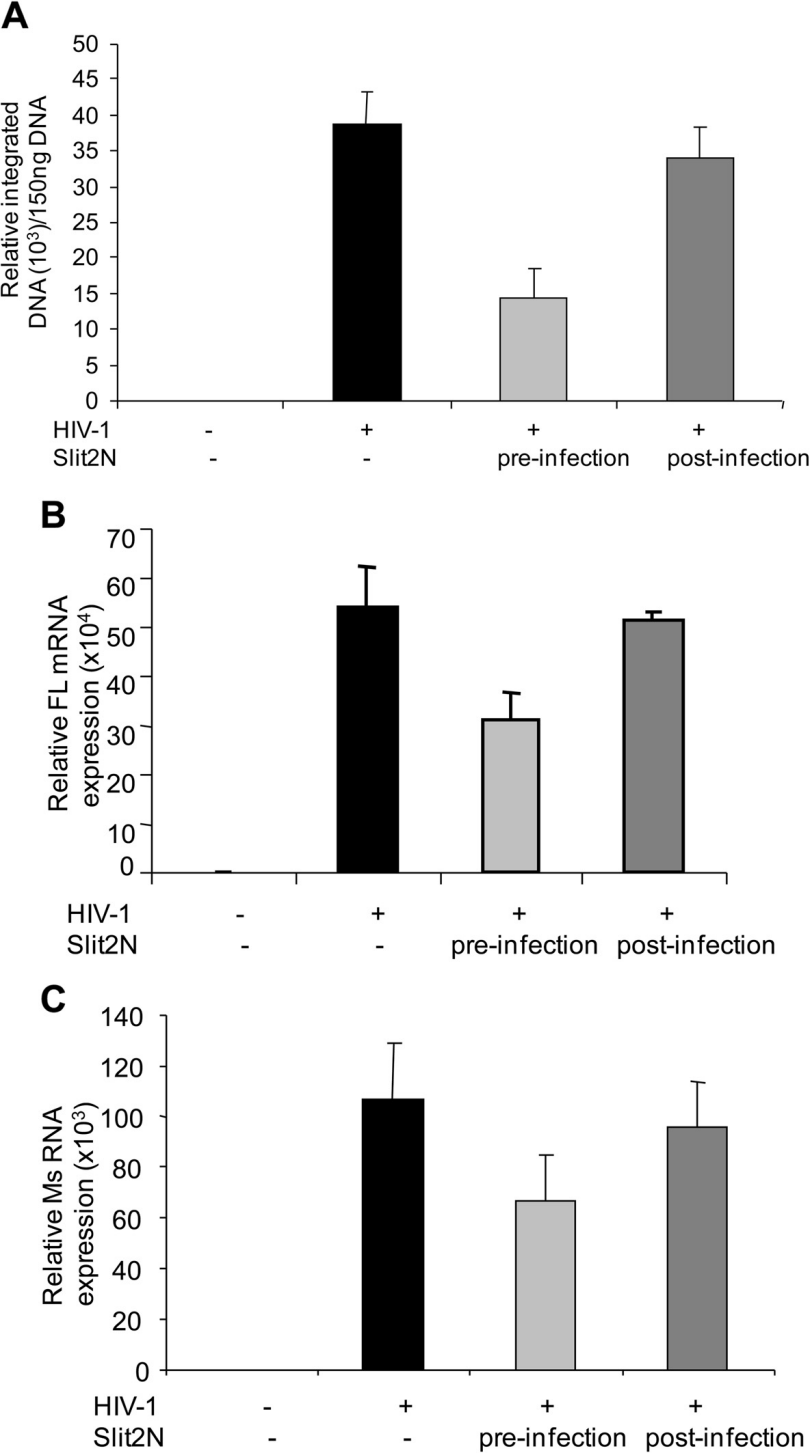
**Slit2N does not affect HIV-1 integration or transcription.** (**A**) MT4 cells were treated with Slit2N prior to HIV-1 infection or three hours after infection (to bypass the entry step). Twenty-four hours after infection, DNA was extracted from the respective samples, and the integrated HIV-1 DNA was analyzed by qPCR for Alu-LTR. (**B**) The full length and spliced (**C**) RNA transcription was evaluated by qRT-PCR. Uninfected and HIV-1 infected MT4 cells were used as controls. Results are representative of 3 separate experiments.

We further evaluated the effect of Slit2N on integration and post-integrative steps including transcription. In one set of samples, we pre-treated MT4 cells with Slit2N prior to infection with HIV-1-IIIB. In another set of samples, we treated the infected cells with Slit2N, 3 hours after infection to bypass the entry step. After twenty-four hours, proviral HIV-1 DNA was measured with qPCR assays toward LTR-Alu sequences (LTR-Alu) as previously described [37], to measure virus integration. We also quantitated the number of full-length and spliced viral transcripts in the infected cells by qRT-PCR. We did not detect a significant difference in virus integration (Figure 4A) or full-length (Figure 4B) and spliced mRNA levels (Figure 4C) between HIV-1 infected untreated MT4 cells and Slit2 -treated MT4 cells, when Slit2N was added after HIV-1 entry step. However, when Slit2N was added before infection of the cells, Slit2N-treated cells showed a significant difference in the both viral integration and transcription. These results suggest that Slit2N does not regulate HIV-1 integration or transcription in T-cells, but inhibits steps prior to integration.

### Anti-HIV-1 activity of Slit2N is mediated through Robo1

**Figure 5:**
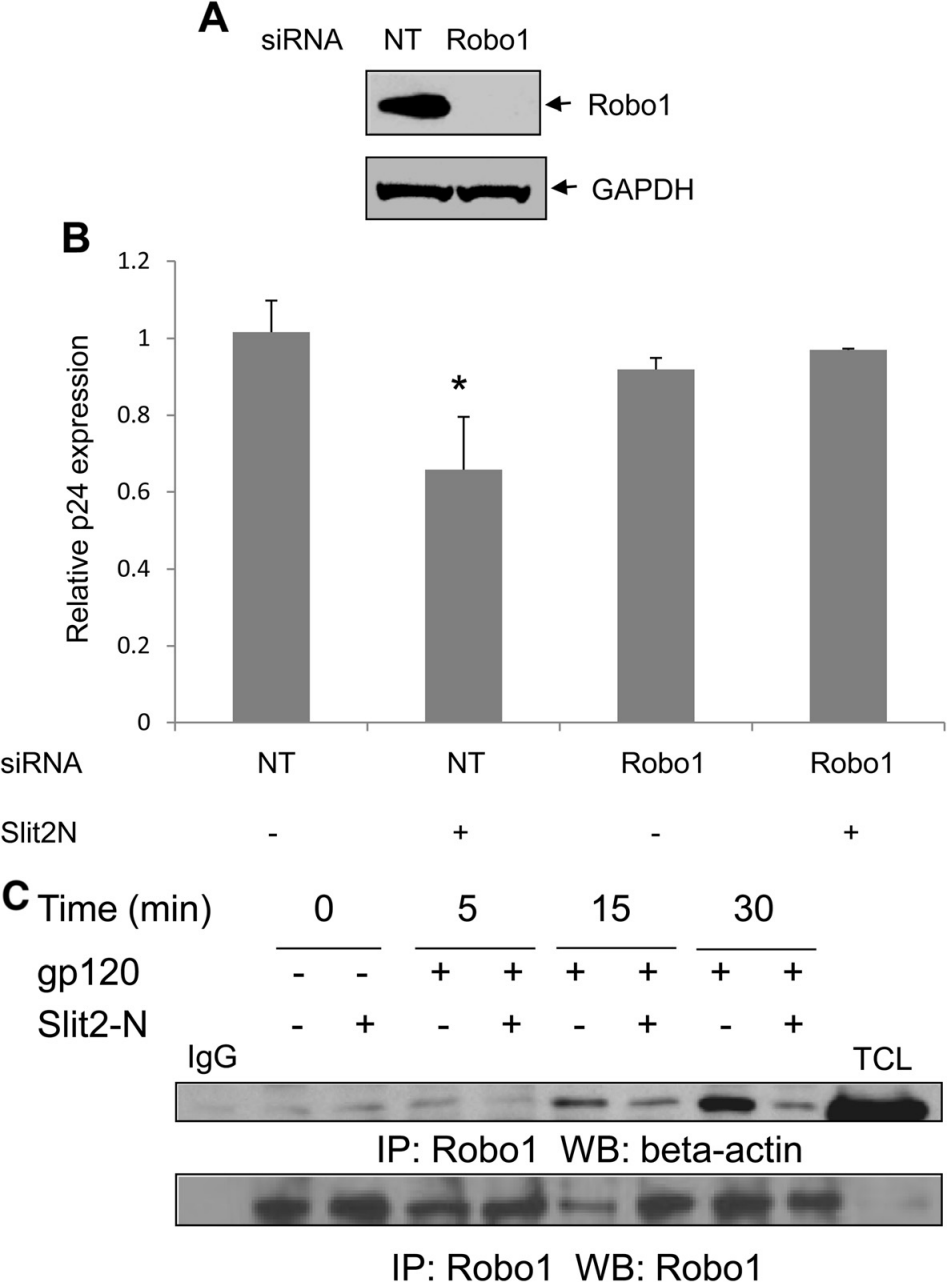
**Slit2N mediates HIV-1 inhibition by binding to the Robo1 receptor.** (**A**) MT4 cells transfected with non-targeting (NT) or Robo- siRNA by nucleofection (Amaxa biosystems) were analyzed for Robo1 expression by Western blotting. GAPDH was used as a loading control. (**B**) The NT and Robo1 siRNA-transfected cells were pre-treated with Slit2N (3nM) or control, and left uninfected or infected with HIV-1 IIIB for 48 hours. The supernatants were analyzed for HIV-1 p24 levels by ELISA. *P < 0.05 versus the mock-infected cells transfected with NT-siRNA. (**C**) MT4 cells were stimulated with HIV-1 gp120 for various periods of time in the presence and absence of Slit2N. The lysates were immunoprecipitated (IP) with anti-Robo1 antibody. The immune complexes were then immunoblotted with β-actin antibody. The same blot was reprobed with Robo1 antibody. TCL- total cell lysate. IgG- antibody control. Results are representative of 3 separate experiments.

Our next aim was to decipher the molecular mechanism by which Slit2N mediates HIV-1 inhibition. Robo1 is a protein with a single transmembrane domain, serving as a Slit2 receptor in various cells and mediates a majority of Slit2N-mediated effects. We, therefore, assessed the role of Robo1 in Slit2N-mediated inhibition of HIV-1 infection. MT4 cells transfected with Robo1 siRNA showed a significant knock-down of Robo1 expression as compared to cells transfected with non-targeting siRNA (Figure 5A). The transfected cells were infected with HIV-1 IIIB and observed for HIV-1 p24 levels after 48 hours of incubation. As shown in Figure 5B, Robo1 downregulation abrogated Slit2-mediated inhibitory effects compared to control, suggesting that the anti-HIV-1 activity of Slit2N is predominantly transduced by the Robo1 receptor.

### Slit2 inhibits the HIV-1-envelope induced Robo1-actin association

Since studies have shown that Robo associates with several actin-related proteins to mediate its functions [38-40], we evaluated whether HIV-1 gp120 regulates the association of Robo1 with actin. Interestingly, we found that treatment with HIV-1 gp120 enhanced the association of the Robo1 receptor with actin. However, pretreatment with Slit2N attenuated the gp120-induced Robo1-actin association at 5, 15 and 30 minute intervals (Figure 5C), indicating that Slit2N upon binding to Robo1 inhibits Robo1/actin association and may thus modulate HIV-1-induced cytoskeletal changes facilitating viral entry.

### Slit2N attenuates HIV-1-envelope-induced actin polymerization in T-cells

**Figure 6:**
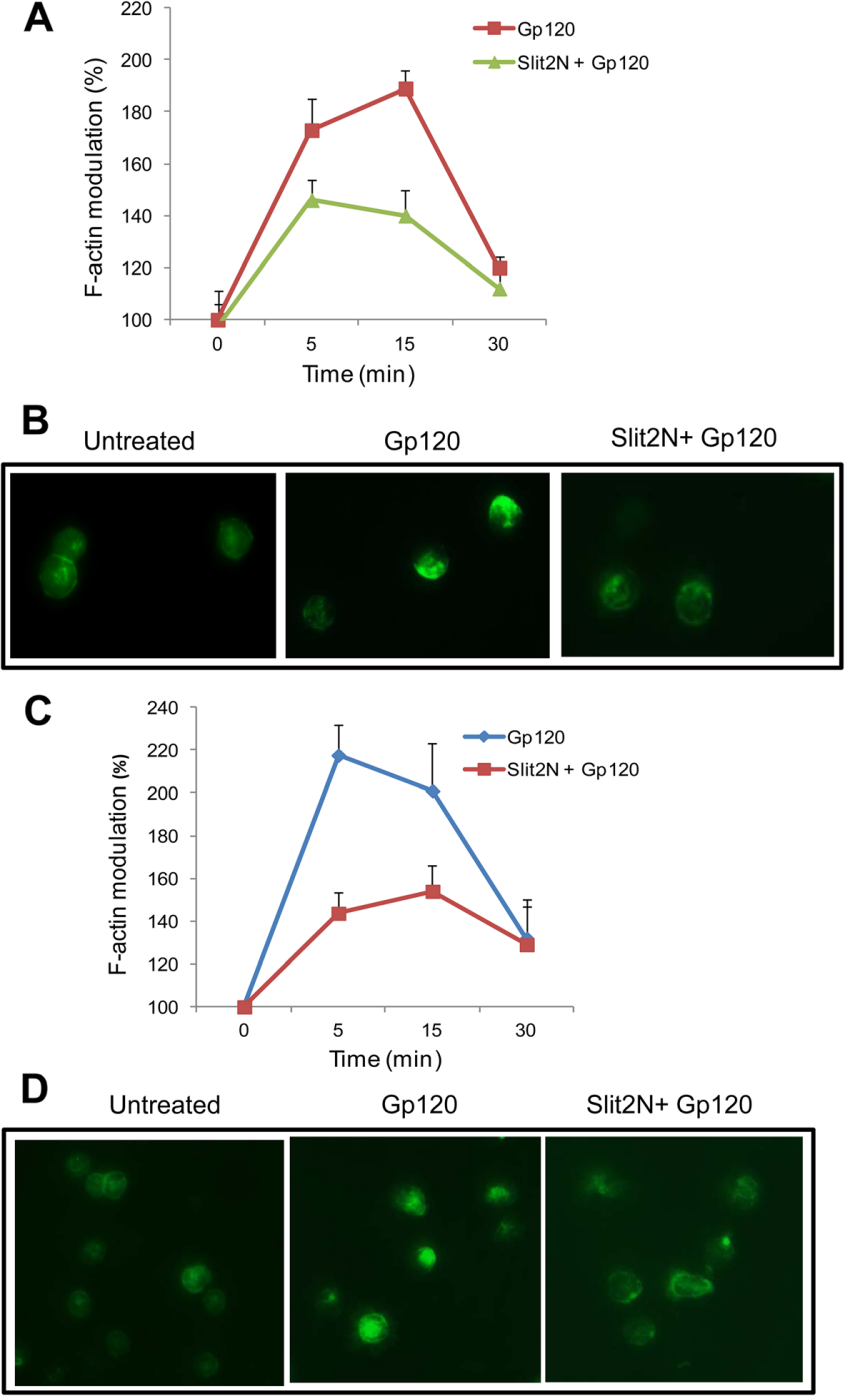
**Slit2N inhibits HIV-1-envelope-mediated actin polymerization.** (**A**) MT4 cells were pre-treated with Slit2N (3nM) and treated with gp120 for various time points, as indicated. Cells were taken at various time points and stained with FITC-phalloidin and quantified by flow cytometry. The graph represents the percentage increase in F-actin positive cells over the control. (**B**) The cells stained with phalloidin-FITC were also observed under a fluorescence microscope. The images shown are cells stimulated with Gp120 for 5 minutes. (**C**) Resting T-cells were pre-treated with Slit2N (3nM) and treated with gp120 for various time points, as indicated. Cells were taken at various time points and stained with FITC-phalloidin and quantified by flow cytometry. The graph represents the percentage increase in F-actin positive cells over the control. (**D**) The cells stained with phalloidin-FITC were also observed under a fluorescence microscope. The images shown are cells stimulated with Gp120 for 5 minutes.

We further investigated the signaling mechanisms of Slit2N-mediated inhibition of viral entry. In HIV-1 infection, binding of the virus to CD4+ T cells initiates an early actin polymerization in T-cells [29,41] followed by depolymerization [29], a process mimicking the chemotactic response initiated from chemokine receptors. The actin polymerization has been suggested to promote viral entry [28-30]. To determine whether Slit2N affects HIV-1-induced cytoskeletal changes, MT4 cells were untreated or treated with Slit2N and stimulated with HIV-1 gp120, fixed and stained for F-actin. As expected, gp120 induced actin polymerization at early time points (15 and 30 minutes). However, in the presence of Slit2N, gp120-induced actin polymerization was significantly inhibited, as demonstrated by flow cytometry (Figure 6A). We also observed a significant inhibition of gp120-induced actin polymerization by Slit2N by fluorescence microscopy (15 minutes) (Figure 6B).

To determine whether Slit2N affects HIV-mediated actin dynamics leading to virus entry in primary cells, we examined the effect of Slit2N on HIV-1 envelope-mediated actin dynamics in resting CD4+ T-cells. Actin polymerization was demonstrated by changes in phalloidin-FITC binding to F-actin over time. We found that HIV gp120 transiently triggered low actin polymerization at early time points in primary T-cells. Pre-treatment with Slit2N inhibited early actin polymerization in these cells (Figure 6C and D), indicating that Slit2 may inhibit HIV-1 entry by modulating the cytoskeletal changes induced by HIV-1.

### Slit2 inhibits signaling mechanisms that regulate Gp120-induced actin cytoskeletal dynamics

We further evaluated the mechanism by which Slit2N inhibits HIV-1 gp120-induced actin polymerization. The mechanism that triggers early actin polymerization through HIV-1 envelope-mediated transient activation of the Rho GTPase, Rac1 and the LIM domain kinase (LIMK), has recently been described [27-29,42,43]. LIMK1 is a protein that phosphorylates and inactivates cofilin that in turn enhances actin polymerization thus facilitating HIV entry. We therefore hypothesized that Slit2N may decrease early actin polymerization by preventing HIV-envelope-induced activation of the Rac1/LIMK1/cofilin pathway.

**Figure 7:**
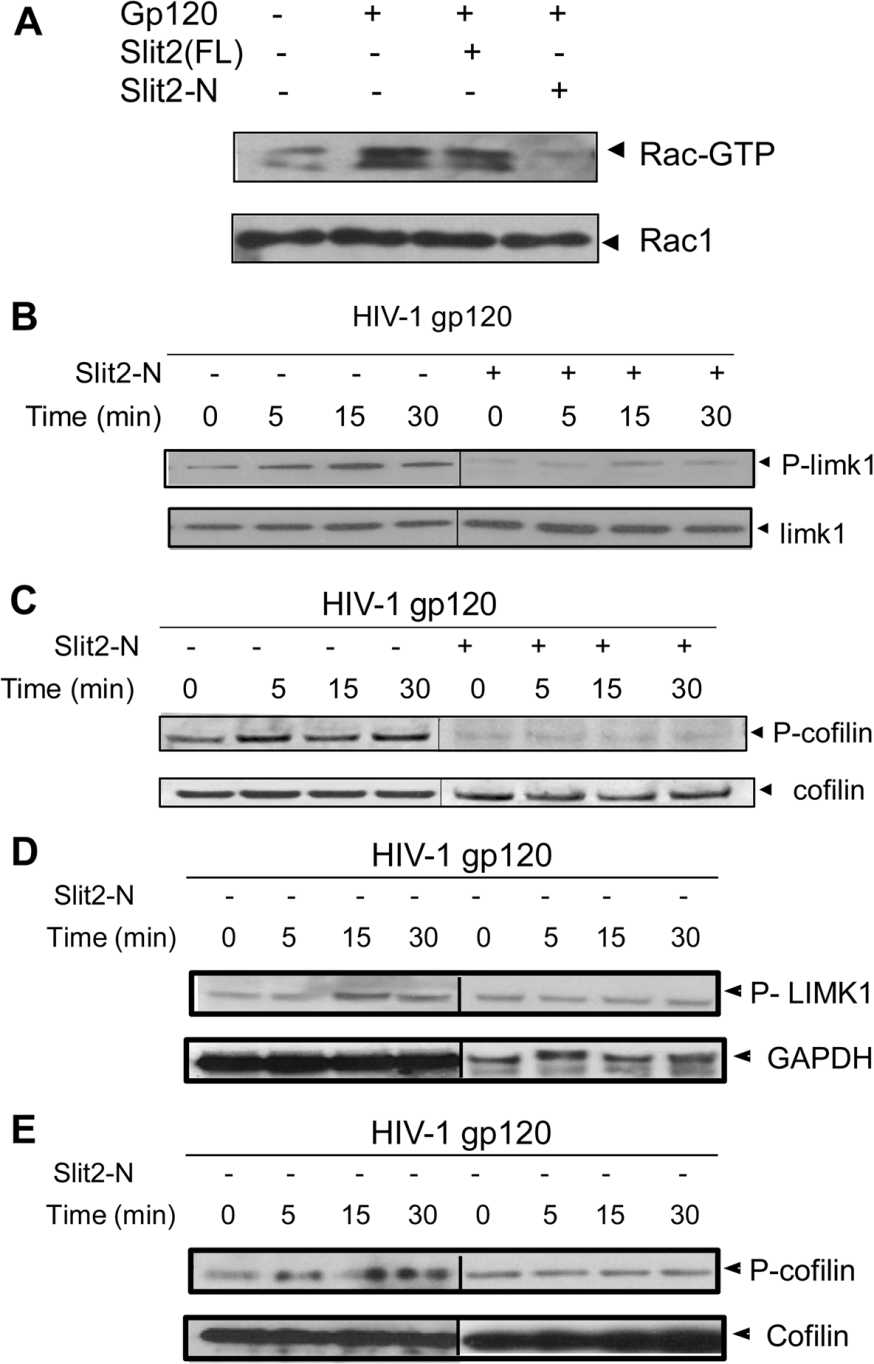
**Slit2N attenuates HIV-1 envelope-induced signaling pathway that mediates actin cytoskeletal reorganization.** (**A**) Lysates from untreated, full-length Slit2 and Slit2N (3nM)-treated MT4 cells were stimulated with X4-tropic HIV-1 gp120 (10nM) for 10 minutes. The lysates were pre-cleared with sepharose beads and incubated with 20 μg/ml p21-activated kinase (PAK)-1 agarose for 60 minutes at 4°C. Agarose beads were collected by centrifugation and analyzed for Rac1-binding activity by Western blotting with anti-human Rac1 antibody (upper panel). Lysates probed with total Rac1 (lower panel) represent loading controls. (**B**) MT4 cells, pretreated with Slit2N, were treated with HIV-1 gp120 for various time points and lysed. To demonstrate LIMK1 activation, the lysates were immunoblotted for phospho-LIMK1. The blots reprobed with total LIMK1 antibody represented loading controls. (**C**) The same lysates were also immunoblotted with phospho-cofilin. Probing for total cofilin represented the loading control. (**D**) Resting primary T-cells, pretreated with Slit2N were treated with HIV-1 gp120 for various time points and lysed. To demonstrate LIMK1 activation, the lysates were immunoblotted for phospho-LIMK1. The blots reprobed with total LIMK1 antibody represented loading controls. (**E**) The same lysates were also immunoblotted with phospho-cofilin. Probing for total cofilin represented the loading control. All of the above experiments were repeated three times, and a representative experiment is shown.

We first tested the effects of Slit2N on HIV-1-envelope-induced Rac1 activation in T-cells. We used agarose beads conjugated to the PBD of PAK (PAK-PBD) to detect the activated, GTP-bound species of Rac1. Unstimulated T-cells had low basal levels of activated Rac1. Exposure to gp120 increased levels of activated Rac1. Slit2N did not affect basal levels of activated Rac1 (data not shown), but inhibited gp120-induced activation of Rac1 significantly (Figure 7A). Furthermore, we found that Slit2N inhibited Gp120-induced phosphorylation of both LIMK1 (Figure 7B) as well as phosphorylation of cofilin (Figure 7C).

We also showed that treatment of resting CD4+ T-cells with Slit2N also interfered with HIV-envelope induced phosphorylation of LIMK1 and cofilin. (Figure 7 D and E). Together, these results suggest that Slit2N inhibits Gp120-induced Rac1/LIMK1/cofilin pathway that mediates actin polymerization, thereby blocking HIV-1 entry.

## Discussion

A prototypical Slit2 protein contains an N-terminal signal peptide, four leucine-rich repeats (LRRs), seven (in *Drosophila* Slit) or nine (in vertebrate Slits) EGF repeats, and a C-terminal cysteine knot [44]. Structural-functional analysis of Slit2 has revealed that the anti-HIV-1 activity of Slit2 was present in the N-terminal domain of Slit2. In the present study, we also showed that the N-terminal region of Slit2 was sufficient to mediate anti-HIV-1 activity in T-cells. A recent study has also evaluated the administration of Slit2N in a murine model of H5N1 influenza [21]. Furthermore, Slit2N also attenuated HIV-1 gp120-induced endothelial permeability [18]. Our findings are thus in agreement with recent studies that indicate that many of the biological functions of Slit2 are mediated by the N-terminal fragment [5,21].

In the present study, we showed that Slit2 not only inhibited infection of T-cells with cell-free virus, but also inhibited cell-cell transmission of HIV-1 using coculture of HIV-1–infected and noninfected cells. Cell-cell transmission of HIV-1, through the formation of a virological synapse, is the predominant mode of HIV-1 transfer *in vivo* and probably occurs in secondary lymphoid organs, where most lymphocytes are present and cells are in close contact with each other and with antigen-presenting cells [34,35].

The initial steps in the HIV-1 viral life cycle include the binding of the viral envelope glycoproteins to the primary receptor, CD4 and co-receptors, CXCR4/CCR5 followed by membrane fusion and viral entry. Recently, Slit2 was shown to downregulate CXCR4 expression in breast cancer cells [8]. However, in contrast to breast cancer cells, Slit2N did not have any effect on the downregulation of either CXCR4/CCR5 or CD4 expression, ruling out down-modulation of HIV-1 receptors/co-receptors as a mechanism of action for Slit2. In addition, virus binding assays indicated that Slit2N did not inhibit HIV-1 binding onto target cells. However, specific entry assays demonstrated that Slit2 inhibited viral entry into the target cell.

In our mechanistic studies, we showed the importance of Robo1 in Slit2-mediated inhibition of HIV-1 replication using siRNA-mediated Robo1 knockdown experiments. This is in agreement with various genetic and biochemical experiments that have shown that Slit2 binds to Robo1 receptor to mediate signaling and many of its functional effects [1,3,4,15,16,23]. Our next aim was to investigate the exact mechanism of inhibition of virus entry. The early interaction between HIV and T cells initiates intracellular signaling cascades that are important for the early steps of the HIV life cycle [28,45].

The cortical actin is a common structure that is targeted by most viruses for entry and intracellular transport [46,47]. In HIV-1 infection, the direct involvement of the cortical actin in early stages of viral infection has been suggested in HIV-mediated CD4-CXCR4 receptor clustering [26,46,48], and intracellular migration [28]. Actin polymerization has been shown to promote viral entry in both the simple model of infection by free virus and the more physiologically relevant route of infection through the virological synapse [26,28-30,45,49]. In this study, we showed that Slit2N inhibits gp120-induced actin polymerization and Robo-actin association at early time points. This is in agreement with the common theme emerging from a number of studies that Slit2 mediates cellular guidance through regulation of actin polymerization as well as data from neuronal cells linking Robo to proteins associated with the actin cytoskeleton including ena and srGAP1 [11,14,22,23,50].

The data implicating that Slit2N inhibits Gp120-induced actin polymerization led us to evaluate the intracellular molecules that transduce the signal to regulate the actin polymerization. Several studies indicate that the regulatory effect of Slit2 involve modulation of Rho family of small GTPases, Rac1 and cdc42 [11,14,23,50]. Rho-GTPases play a definitive role in cell migration, cytoskeletal organization and actin remodeling. A recent study has also shown that the binding of HIV-1 envelope glycoprotein with the primary receptor CD4 and one of the co-receptors, CXCR4 or CCR5 activates a signaling cascade resulting in activation of Rho GTPases, specifically Rac1 and actin cytoskeletal remodeling that facilitate HIV-1-induced membrane fusion and virus entry [27]. Rac1 has been shown to be activated by gp120 to trigger actin polymerization to mediate both cell-cell [49] and virus-cell fusion [27]. Furthermore, another recent study has described a pathogenic mechanism for triggering early actin polymerization through HIV-1 envelope-mediated transient activation of Rac1-LIMK1-cofilin pathway [29].

**Figure 8:**
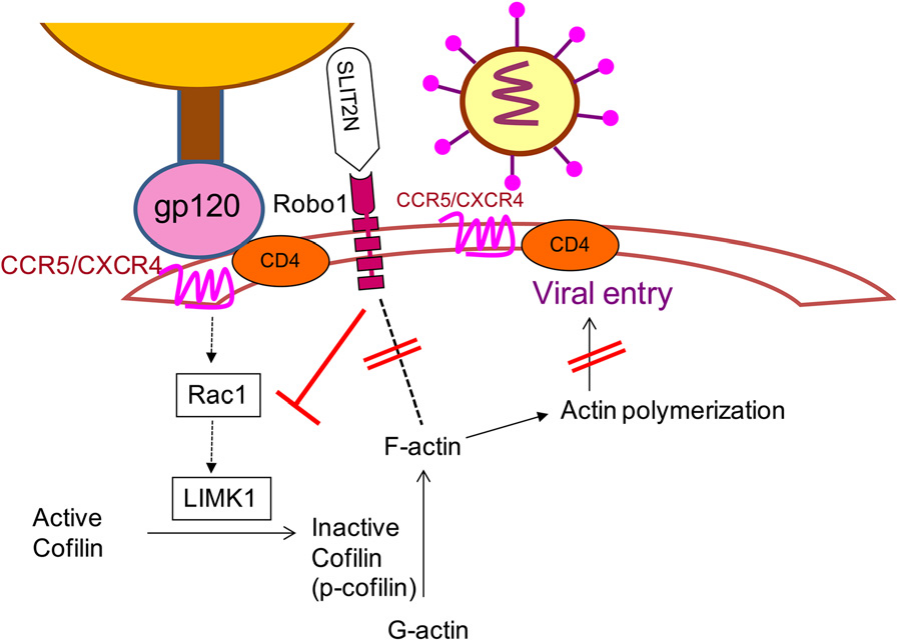
**Proposed mechanism of Slit2N-mediated inhibition of HIV-1 entry.** Slit2N inhibits HIV-1 envelope-induced Rac1/LIMK1/cofilin pathway, thus attenuating actin polymerization, ultimately blocking virus entry. Furthermore, Slit2N also inhibits HIV-1 envelope-induced Robo1/actin association, possibly modulating cytoskeletal rearrangements that facilitate HIV-1 entry.

The LIM domain kinase, LIMK, is a protein that phosphorylates cofilin and LIMK1-mediated actin polymerization was shown to directly facilitate early CD4 CXCR4 clustering and viral entry. These studies led us to test the effects of Slit2N on the HIV-1-envelope-induced activation of LIMK1 and cofilin. We found that Gp120-induced phosphorylation of LIMK1 and cofilin was inhibited by Slit2N. Previously, it has been shown that early LIMK1 activation in response to HIV envelope occurs in both unstimulated and stimulated T-cells [29]. Furthermore, the authors also showed LIMK activation upon infection of human primary macrophages with the CCR5-utilizing viruses [29]. These findings indicate that the Rac1/LIMK1/cofilin pathway leading to early actin polymerization is rather a general mechanism occurring early in both X4 and R5-tropic HIV infection of both primary resting/active CD4 T cells and macrophages. Based on our results, we hypothesize that Slit2N blocks HIV-1 induced activation of Rac1/LIMK1/cofilin pathway that leads to actin polymerization, thus blocking HIV-1 entry (Figure 8).

## Conclusion

In summary, our studies reveal that N-terminal region of Slit2 possesses anti-HIV-1 activity against both X4 and R5-tropic viruses. Furthermore, elucidation of the signaling mechanisms revealed that Slit2N regulates HIV-1 infection by inhibiting HIV entry into target cells through modulation of cytoskeletal dynamics. We have shown that Slit2N inhibits gp120-induced Robo1-actin association. In addition, our studies revealed that Slit2/Robo1 modulates cytoskeleton by attenuating Rac/LIMK1/cofilin pathway. Our findings thus elucidate the role of Slit2N in HIV-1 infection and could provide insights into novel approaches to limit HIV-1 infection.

## Methods

### Viral isolates

The lab-adapted X4-tropic virus, HIV-1 IIIB and the R5-tropic virus, HIV-1 BaL, used in this study were obtained from the NIH AIDS Research and Reference Reagent Program.

### Anti-HIV-1 assays

MT4 cells, a human T-cell line bearing human T-cell leukemia virus type 1 (NIH AIDS Research and Reference Reagent program) were grown in RPMI 1640 medium (Mediatech, Manassas, VA) supplemented with 10% heat-inactivated fetal calf serum (FCS) (Invitrogen, Carlsbad, CA) and antibiotics. Peripheral blood mononuclear cells (PBMCs) were isolated from heparinized venous blood collected from healthy HIV-1-seronegative donors (American Red cross, Columbus, OH) by Ficoll-Paque density gradient centrifugation (GE Biosciences, Piscataway, NJ). The study was done in compliance with the Helsinki declaration on rights of human subjects with approval from the Ohio State University Institutional Review Board (2007H0281). Monocytes were depleted by two rounds of adherence to plastic. Nonadherent cells were either rested overnight in complete RPMI and used for infection or stimulated with phytohemagglutinin (PHA) (5 μg/ml) for 3 days. CD4^+^ T-cells were isolated according to the manufacturer’s protocol by immunomagnetic selection (EasySep Kit, Stem Cell Technologies, Canada).

MT4 cells pre-treated with various concentrations of Slit2N (0.6-60 nM) were infected with HIV-1 at 10 ng p24/10^6^ cells. The cells were washed extensively after two hours of infection to remove the unbound virus and incubated for 48 hours. PHA-stimulated PBMCs were pre-treated with Slit2N and infected similarly with HIV-1. The culture supernatant were assessed with the HIV-1 p24 ELISA (Advanced Bioscience Laboratories, Kensington, MD).

For infection of resting CD4+ T-cells, HIV-1 IIIB (10 ng p24) was used to infect 10^6^ cells. For these studies, T-cells were pretreated with Slit2N (6nM), incubated with the virus for 2 hours at 37°C and washed twice with medium to remove the unbound virus. Infected cells were resuspended into fresh complete RPMI containing Slit2N (6nM) at a density of 10^6^/ml and incubated for 5 days without stimulation. Cells were activated at day 5 with anti-CD3/CD28 magnetic beads (Dynal) at 2–4 beads per cell to initiate viral replication. Infected cells were pelleted at various time points and supernatants used for p24 ELISA.

### Quantification of cell-cell spread by flow cytometry

To measure cell-cell spread by flow cytometry, an adaptation of the assay of Sourisseau *et al*. [51] was used. Briefly, an equal number of HIV-1-infected donor MT4 cells were mixed with CellTrace Far Red-labeled target T cells (2 μM; Invitrogen) in the presence of various concentrations of Slit2N (0–60 nM) and incubated for 4 h at 37°C. In addition, infected cells were treated with trypsin-EDTA prior to fixing to further diminish doublet formation and to remove surface-absorbed p24. Cells were then fixed in 4% formaldehyde, washed, and permeabilized in BD Perm/Wash buffer (BD Biosciences), and HIV-1 p24 was detected with the FITC-conjugated HIV p24 antibody (Pierce) and data acquired using a FACSCalibur (BD Biosciences). The percentage of HIV-p24^+^ cells among the CellTrace FarRed-labeled target cells was quantified and data analysis performed using CellQuest 5.0.

### Proteins antibodies and plasmids

Slit2N protein was obtained either from AbCam (Cambridge, MA) or Creative Biomart (Shirley, NY); LIMK1, Cofilin and GAPDH antibody was obtained from Cell signaling (Danvers, MA), while Robo1 antibodies were obtained from AbCam. Slit ΔD2 was prepared by transfecting the Slit ΔD2 plasmid in 293 T cells. The protein was purified from the supernatants as described before [16]. The Slit ΔD2 plasmid was cloned in pTT28 by deleting the second LRR domain of truncted Slit2 (Slit2-N) using restriction enzymes BsrGI and NheI. After deleting 210 amino acids (Q235-W444) of Slit2-N, a short synthesized linker corresponding to amino acids YTAGGSAGGSAGGSAGKL was inserted into BsrGI and NheI restriction sites [33].

### Preparation and infection of HIV-1 pseudotyped viruses

HIV-1 viruses pseudotyped with vesicular stomatitis virus envelope protein (VSV-G) were prepared as previously described [52,53]. Briefly, 293 T cells (27 × 10^6^ per 10 cm plate) were transfected with 20 μg of pNL4-3.Luc.R-E- (HIV-Luc) plasmid and 4 μg of pVSV-G (obtained from the NIH AIDS Research and Reference reagent program) by lipofectamine 2000 reagent (Invitrogen) according to the manufacturer’s instructions. Cell culture supernatants were collected 48 hours later, filtered and saved as virus stocks. For infection, pseudotyped viruses corresponding to 10 ng p24 were used to infect target cells. Following 2 hour infection, the cells were washed extensively to remove unbound viruses. The cells were incubated for 48 hours and harvested for luciferase activity as previously described [53].

### Immunostaining and flow cytometry

FITC- or PE-labeled monoclonal antibodies against human CD4, CCR5 or CXCR4 were purchased from BD Biosciences. Half million cells were incubated with isotype control or the labeled antibodies on ice in PBS-0.1%BSA for 30 minutes. Cells were washed with cold PBS-0.5% BSA, and then analyzed on a FACSCalibur (BD Biosciences). To determine intracellular p24, HIV-1 infected T- cells were fixed and permeabilized using Fix/perm solution (BD Biosciences, San Jose, CA) and stained with KC57 monoclonal antibody (Coulter, Brea, CA) followed by flow cytometry analysis. The respective isotype control was also included. The flow cytometry data was analyzed by CellQuest software (BD Biosciences).

### Gp120 binding assay

MT4 cells were incubated for 1 hour at 37°C with differing concentrations of Slit2N After the incubation, cells were washed with PBS and incubated with 1 μg/mL FITC-conjugated recombinant gp120 (Immunodiagnostics, Woburn, MA) for 30 minutes at room temperature. The fluorescence intensity of gp120-FITC bound to the surface of lymphocytes was measured with the FACS Calibur.

### Virus binding assay

The procedure used to detect the binding of HIV-1 particles into target cells was described previously [54]. Briefly, MT4 cells (1.0 × 10^6^ cells/mL) were exposed to HIV-1 (40 ng p24) in the absence or presence of the Slit2N (3 and 6 nM) in 100 μL of PBS. After incubation at 4°C for 1 h, unbound virus particles were removed by washing the cells three times in PBS. Cell lysates were prepared by resuspending the pellet in lysis buffer (PBS containing 1% Triton X). Viral binding was monitored by measuring the amount of p24 in the cell lysates by ELISA. Total cell protein was calculated using Bradford assay and all samples were normalized for protein content.

### Viral entry assays

In the first method, MT4 cells (1.0 × 10^6^ cells/mL) were exposed to HIV-1 (40 ng p24) in the absence or presence of the Slit2N (3nM and 6 nM) in 100 μL of PBS. After incubation at 37°C for 4 hours, unbound virus particles were removed by washing the cells three times in PBS. The cells were treated with 0.05% trypsin for 10 minutes to remove surface-bound viral particles. Cell lysates were prepared by resuspending the pellet in lysis buffer (PBS containing 1% Triton X). Viral entry was monitored by measuring the amount of p24 in the cell lysates by ELISA. Total cell protein was calculated using Bradford assay and all samples were normalized for protein content.

In the second approach, viral entry was measured by the virion-based fusion assay [36]. Briefly, a total of 1 x 10^6^ MT4 cells were incubated 3 h with equivalent viral inputs of BlaM-Vpr-containing virions (500 ng of p24) in 500 μl RPMI 1640 medium. Cells were then extensively washed to remove free virions and incubated (1 h, room temperature) with CCF2-AM loading mix, as recommended by the manufacturer (GeneBLAzer detection kit; Invitrogen). Next, excess dye was washed off and cells were incubated for 16 h at room temperature before fixation with 4% paraformaldehyde. Then, 8 × 10^5^ cells were placed in a 96-well plate per each experimental condition. The associated emission light to cleaved CCF2 (blue; 447 nm) and intact CCF2 probe (green; 520 nm) was measured. The entry of BlaM-Vpr containing virions was measured as the ratio of the maximum fluorescence intensity between cleaved and intact CCF2. Thereby, an increase in this ratio indicates more fused viruses with target cells. The percentage of infection was determined by measuring the fluorescence intensities of intact and cleaved CCF2 probe in control infected cells (scrambled or pCDNA.3 transfected cells) and subtracting the background blue and green fluorescence ratio determined in noninfected cells (without β-lactamase activity), (GeneBLAzer detection kit; Invitrogen).

### Virus integration

Host cell nuclear integration of HIV in MT4 cells was analyzed by PCR as described previously [55]. DNA was prepared by using a DNA extraction kit (Qiagen, Valencia, CA) according to the manufacturer’s protocol. DNA samples were used for the amplification of integrated DNA with Alu-LTR–specific primers [37] using the following cycling conditions: 95°C for 10 minutes, then 40 cycles at 95°C for 15 s, 60°C for 1 minute, and 72°C for 1 minute. GAPDH was used as an internal control.

### Determining proviral transcription of full-length (FL) and multiple-spliced (MS) HIV-1 mRNA

RNA was prepared from untreated or Slit2N treated HIV-1 IIIB-infected cells using the QIAamp RNAeasy Mini Kit protocol (Qiagen). FL and MS HIV-1 mRNA transcript levels were determined by a modification of a protocol described [56]. qRT-PCR was performed using primers (200nM) specific for FL and for MS using 400 ng of sample RNA in a SYBR Green assay system using a Realplex Cycler (Eppendorf, Westbury, NY).

### Transfections

SiRNA-mediated knockdown of Robo1 was performed using specific ON-TARGETplus SMARTpool siRNA (Dharmacon, Lafayette,CO). Briefly, MT4 cells were nucleofected with 200 nM siRNA (Amaxa Biosystems). The respective, non-targeted (NT) siRNA was used as a control. Robo1 siRNA- -mediated knockdown was estimated by detecting Robo1 expression 48 hours after the initial transfection by Western blotting and/or flow cytometry.

### Rac1 activation assay

Rac1 activation was determined by using the Rac/Cdc42 activation assay kit (Millipore). In brief, cell lysates were pre-cleared with sepharose beads were incubated with 20 μg/ml p21-activated kinase (PAK)-1 agarose for 60 minutes at 4°C, according to the manufacturer’s instructions. Agarose beads were collected by centrifugation, followed by denaturation, boiling of the samples, and SDS-PAGE analysis. Proteins were transferred to nitrocellulose membranes, and Western blotting was performed by using murine anti-human Rac1 antibody. Blotting of equal amounts of lysates with total Rac1 represented the loading controls.

### FITC-Phalloidin staining of F-actin

One to two million cells were stimulated with HIV-1 gp120 IIIB and incubated at 37°C with gentle agitation (600 to 1200 rpm) to prevent cell settling at the bottom. F-actin staining using FITC-labeled phalloidin (Sigma, St. Louis, MO) was carried out according to the manufacturer’s recommendation with minor modifications. Briefly, each staining was carried out using 1–2 × 10^6^ cells. Cells were pelleted, fixed and permeabilized with CytoPerm/Cytofix buffer (BD Biosciences) for 20 minutes at room temperature, washed with cold Perm/Wash buffer (BD Biosciences) twice, followed by staining with 5 μl of 0.3 mM FITC-labeled phalloidin for 30 minutes on ice in dark. After washing twice with cold Perm/Wash buffer, cells were resuspended in 1% paraformaldehyde and analyzed on a FACSCalibur (BD Biosciences). The cells were also observed under a fluorescence microscope (Zeiss Axiophot). Cells were scored as positive if increased F-actin staining was observed.

### Western blotting

Equivalent amounts of protein extracts were run on a 4% to 12% gradient acrylamide gel (NuPAGE Bis-Tris gel; Invitrogen) and transferred onto nitrocellulose membranes. Immunodetection involved specific primary antibodies, appropriate secondary antibodies conjugated to horseradish peroxidase, and the enhanced chemiluminescence Western blotting detection system (GE Lifesciences).

### Statistical analysis

Reported data are the means ± S.E.M. of at least three independent experiments performed in duplicate or triplicate. The statistical significance was determined by the Student’s *t* test.

## Competing interests

The authors declare that they have no competing interests.

## Authors’ contributions

ARA designed and performed experiments, analyzed data and wrote the manuscript. HZ performed experiments, analyzed data and wrote the manuscript, TN performed the experiments and analyzed the data, LAR designed and contributed an important reagent for the experiments and RKG conceived the study, designed experiments, analyzed data and wrote the manuscript. All authors read and approved the final manuscript.
